# Realities and hopes in the application of microbial tools in agriculture

**DOI:** 10.1111/1751-7915.13866

**Published:** 2021-06-22

**Authors:** Bruna D. Batista, Brajesh K. Singh

**Affiliations:** ^1^ Hawkesbury Institute for the Environment Western Sydney University Richmond NSW Australia; ^2^ Global Centre for Land‐Based Innovation Western Sydney University Richmond NSW Australia

## Abstract

The use of microbial tools to sustainably increase agricultural production has received significant attention from researchers, industries and policymakers. Over the past decade, the market access and development of microbial products have been accelerated by (i) the recent advances in plant‐associated microbiome science, (ii) the pressure from consumers and policymakers for increasing crop productivity and reducing the use of agrochemicals, (iii) the rising threats of biotic and abiotic stresses, (iv) the loss of efficacy of some agrochemicals and plant breeding programs and (v) the calls for agriculture to contribute towards mitigating climate change. Although the sector is still in its infancy, the path towards effective microbial products is taking shape and the global market of these products has increased faster than that of agrochemicals. Promising results from using microbes either as biofertilizers or biopesticides have been continually reported, fuelling optimism and high expectations for the sector. However, some limitations, often related to low efficacy and inconsistent performance in field conditions, urgently need to be addressed to promote a wider use of microbial tools. We propose that advances in *in situ* microbiome manipulation approaches, such as the use of products containing synthetic microbial communities and novel prebiotics, have great potential to overcome some of these current constraints. Much more progress is expected in the development of microbial inoculants as areas such as synthetic biology and nano‐biotechnology advance. If key technical, translational and regulatory issues are addressed, microbial tools will not only play an important role in sustainably boosting agricultural production over the next few decades but also contribute towards other sustainable development goals, including job creation and mitigation of the impacts of climate change.

## Graphical abstract

Advances in *in situ* microbiome manipulation approaches, such as the use of products containing synthetic microbial communities and novel prebiotics, have the potential to overcome major limitations in the development of microbial tools in agriculture. Much more progress is expected in the development of microbial inoculants as areas such as synthetic biology and nano‐biotechnology advance.

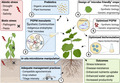

## Major drivers for the development of microbial tools in agriculture

One of the main challenges that agriculture faces in the 21st century is to sustainably produce enough food, fibre and biofuel to meet the needs of a rapidly growing population (FAO, 2017). The availability of arable land and water resources has declined, but the threats of biotic and abiotic stresses, mainly induced by the changing climate, have increased (Pandey *et al*., 2017). This has resulted in further intensification of the use of chemical fertilizers and pesticides aiming to increase crop productivity. However, the environmental and economic costs of applying these agrochemicals to crops are often high. Various reports have shown that the prolonged use of agrochemicals may lead to soil degradation, loss of biodiversity, water pollution, induction of pests/diseases resistance and adverse impacts on human health, among many other negative effects (Sud, 2020). This has led to strong demand from society and regulators for reduced use of chemicals in agriculture. In addition, the loss of efficacy of some agrochemicals and plant breeding programs and the structural decline in soil fertility mean that further addition of chemical inputs does not translate into a proportional increase in crop productivity.

The use of plant growth‐promoting microbes (PGPMs) for plant nutrition and protection is increasingly considered an environmentally responsible complement/alternative to agrochemicals with the potential benefits of addressing the twin global challenges of food security and environmental sustainability (Singh and Trivedi, 2017; Lopes *et al*., 2021). PGPM are free‐living or symbiotic bacteria and fungi that successfully colonize the plant host and exert beneficial effects on its development. The benefits that PGPMs exert to the plant growth can be direct or indirect. Direct benefits include the facilitation of essential nutrient acquisition, provision of phytohormones and inhibition of plant pest and pathogens. Indirect benefits are usually related to changes in the plant physiology and immune system to alleviate the effects of biotic and abiotic stresses (Trivedi *et al*., 2020). Although PGPMs naturally occur in the rhizosphere, phyllosphere and endosphere of plants, their populations are often insufficient to obtain the desired effect. Thus, these microorganisms are usually isolated from their original environment, multiplied and reintroduced as microbial inoculants (or plant probiotics) into the soil or into the plant via seed treatment, foliar spray or soil application (Naamala and Smith, 2020; French *et al*., 2021).

## The golden decade for microbiome research and development

The benefits of microbial inoculation have been known for over 120 years. In 1896, the product ‘Nitragin’ containing a nitrogen (N)‐fixing *Rhizobium* spp. became the first patented bioinoculant in the USA (Arora *et al*., 2017). In the 1950s, initial studies on arbuscular mycorrhizal fungi reported promising effects of these inoculants on increasing P uptake and plant growth (Koide and Mosse, 2004). At the same time, the fertilizer ‘phosphobacterin’ containing kaolin rocks and spores of *Bacillus megaterium* var. *phosphaticum* was used in the Soviet Union, increasing the crop yield by up to 70% (Ribeiro *et al*., 2020). Despite these success stories, the adoption of such technologies by the manufacturing industry and global farming community remained insignificant for a long time. This is probably due to technical limitations that prevented advances in the development of microbial products. For many years, microbiology science relied on culture‐dependent methods, which require the cultivation of the microorganisms in standard culture media. But culturable microbes constitute only 1% of the total microbial community, and the huge potential of the unculturable microbes has remained untapped (Singh, 2010).

Over the last 10 years, there has been a steady emergence of technologies to access and study both culture‐dependent and ‐independent microbial communities (i.e. the microbiomes). High‐throughput sequencing and new tools for analysing metagenomic data have allowed researchers, for the first time, to investigate the composition and ecologic aspects of the microbiomes (Waldor *et al*., 2015). Consequently, the interdependence between plants and their associated microbiomes and the impact of these microbiomes on the host’s fitness and productivity has become clear.

New knowledge led to increased enthusiasm to develop and use microbial tools in agriculture. The recent mapping analysis carried out by Canfora *et al*. (2021) provides evidence of the renewed interest in the sector. The study assessed trends in research on microbial inoculants over time and revealed that fewer than 100 articles were published before 2000 on the topic. From 2000 to 2020, a total of 682 scientific publications were found. Almost 54% of these total publications were published between the years 2015 and 2020, that is, in the last 5 years. The authors also reported that China, India, the USA and Germany are the current leaders in microbial inoculants research worldwide, accounting together for almost 40% of the publications in the area over the last 20 years.

Due to the high application potential of microbial products and the valuable tools emerging recently, major investments in this sector have been made, and the expectations have been high (Sessitsch *et al*., 2018). The global market value of agricultural microbes is estimated to reach around USD 12 billion by 2027, which is roughly triple the current value (~ USD 4.5 billion) (Fortune Business Insights, 2021). In the last few years, the number of start‐ups developing and commercializing microbial products has increased significantly. AgBiome, BioInnovations, Indigo, Maronne and New Leaf Symbiotics are a few examples. In addition, the world’s largest agriculture/food companies have been investing heavily in biological solutions, betting on microbes as the tool for the future of agriculture. Particularly from 2012, many acquisitions, licencing agreements and partnerships worth hundreds of millions of dollars show the depth and breadth of investments that large companies have been making in the sector (Olson, 2015). BASF SE (Germany), E.I. DuPont de Nemours and Company (US), Bayer Crop Science (Germany) and Novozymes A/S (Denmark), along with Verdesian Life Sciences, LLC (US), are currently the top five microbial inoculants companies in the global market (Sammauria *et al*., 2020).

Recent policy changes have also boosted the market for microbial products worldwide. For instance, the new Green Deal of the European Union aims to reduce the use of N‐fertilizer by at least 20% and the use of chemical pesticides by 50% by 2030 (European Commission, 2020). The reduction in agrochemicals usage will require replacement by sustainable tools such as the microbial products. The EU policymakers will likely favour the commercialization of food products grown in regions that use sustainable farming practices. In the medium term, this will have a cascading effect on policies in other parts of the globe through trade, given that EU is a dominant importer of food.

## Biofertilizers and biopesticides market

Commercially, products formulated with PGPMs are usually referred to as biofertilizers and biopesticides. PGPMs act as biofertilizers when they increase the availability of key nutrients, such as nitrogen (N) and phosphorous (P), to the plant. For this, the microbes in the inoculants employ biological N fixation (BNF) and phosphate solubilization/mineralization. PGPMs may also directly promote plant growth by providing or altering the metabolism of phytohormones, such as auxins, cytokinins, abscisic acid, gibberellins and ethylene (Lopes *et al*., 2021). Currently, N‐fixing inoculants make up 79% of the global biofertilizer market, with a current market value of about USD 1.5 billion, which is estimated to double by 2024. North America (the USA, Canada and Mexico) holds the largest share in the global biofertilizer market in terms of production, accounting for around 27.7%. However, Europe and Latin America are currently the top consumers of biofertilizers, followed by China and India (Soumare *et al*., 2020).

Biopesticides are PGPMs, or compounds derived from PGPMs, that act as biological control agents (BCAs) by suppressing or controlling pests or diseases. These BCAs may induce plant resistance, compete with pathogens for nutrients and space or employ hyperparasitism or antibiosis against bacterial and fungal pathogen cells (Köhl *et al*., 2019).

The biopesticides global market is currently valued at about USD 3 billion, which accounts for only 5% of the chemical pesticides market. However, some predict that the market of biopesticides will equalize with that of chemical pesticides between the late 2040s and the early 2050s as it grows at a faster pace (Olson, 2015; Singh, 2017, 2017; Damalas and Koutroubas, 2018; Akutse *et al*., 2020). North America represents the largest biopesticides market (44%), followed by the European Union (20%), Oceania (20%), Latin and South American countries (10%) and Asia (~ 6%) (Bailey *et al*., 2010). At present, these products have been gaining increasing interest as an important component of integrated pest management (IPM) programs.

## Stories of success and market trends

### Biofertilizers

Soybean is probably the most successful case of a crop benefiting from the application of microbial inoculants. South American countries such as Brazil and Argentina lead in soybean inoculation (Santos *et al*., 2019). In Brazil, the inoculation of soybean with elite N‐fixing *Bradyrhizobium* spp. strains can fully supply the crops’ N demand, eliminating the need of N‐fertilizers in soybean. This saves the country about USD 13 billion per year in terms of N‐fertilizer equivalents (Zilli *et al*., 2021) and contributes towards climate change mitigation by significantly reducing the emission of greenhouse gases (GHGs), such as nitrous oxide (N_2_O). In the 2019/2020 crop season, 70 million doses of inoculants for soybean were commercialized in Brazil, which covers approximately 78% of the cropping area (about 36.5 million hectares). On the other hand, it is estimated that only 15% of the soybean cropped area in the USA uses N‐fixing microbes (Santos *et al*., 2019). This discrepancy is likely due to the low cost of N fertilizers in the USA, as well as the lack of regulatory support in the country. Therefore, the use of N‐fixing inoculants for soybean in Brazil is a good example of how the benefits of using microbial tools in agriculture go beyond productivity gains: There is a simultaneous reduction in the environmental footprints of agrochemicals, such as GHG emissions and water pollution.

The farm success of soybean inoculation with *Bradyrhizobium* spp. resulted in increased demand for microbial inoculants for other crops, especially corn (*Zea mays* L.) and wheat (*Triticum aestivum* L.) (Santos *et al*., 2021). In this context, significant attention has been focused on some PGPM representatives of the genus *Azospirillum*, such as *A. lipoferum* and *A. brasilense*. These strains, unlike *Rhizobium* strains, are not limited by host specificity. They promote the growth of a wide variety of crops mainly by producing phytohormones and by supplying N through non‐symbiotic BNF (Cassán *et al*., 2020; Raffi and Charyulu, 2021). In 1996, Argentina was one of the first countries to release a commercial product named Nodumax‐L^®^ (Laboratorios Lopez SRL) containing an *A*. *brasilense* strain. In 2009, the first *Azospirillum*‐based product, the Masterfix L Gramineas^®^ (Stoller do Brasil SA) was commercialized in Brazil (Cassán *et al*., 2020). One decade later, Brazilian farmers applied about 10.5 million doses of inoculants containing *Azospirillum* spp. in grasses, such as corn, wheat, rice and pasture, and in legumes such as soybean and common bean (Santos *et al*., 2021).

### Biopesticides

The most successful examples of microbes used as biopesticides include the entomopathogenic *Bacillus* *thuringiensis* (Bt), *Pseudomonas* spp., baculoviruses, *Beauveria* spp., *Metarhizium* spp. and the mycoparasite *Trichoderma* spp. Presently, about 75% of the commercial biopesticides consist of products derived of Bt (Samada and Tambunan, 2020). These products were commercially produced in France in 1938 and in the USA in 1956, but their use increased worldwide in the 1980s, when insects became increasingly resistant to chemical insecticides (Abbas, 2018). Bt‐derived products are now marketed for the control of different important plant pests, including caterpillars, beetles, mosquito larvae and black flies (Patel and Rahul, 2020). Moreover, toxin genes from Bt have been genetically engineered into several crops, such as cotton, corn and potato (Kumar *et al*., 2020) with significant commercial success. This demonstrates the potential of these microbes not only in directly protecting plants against diseases and pests but also as a reservoir of novel genes of interest to genetically modify crops for increased productivity.

The crop protection market, which has relied on agrochemicals for a long time, has been increasingly affected by several factors such as the decline of new active ingredients entering the market, the resistance development in pathogens and pests against existing chemicals, the use of genetically modified seeds solutions and the rising costs and requirements of regulatory bodies for agrochemicals. This is further complicated by the pressure from society and regulators for reduced chemical residues in the food and environment, the low margins of farm profitability, the adoption of integrated pest management in many places and the increasing opportunities in the organic food sector (Sessitsch *et al*., 2018; Phillips, 2020). The search for alternative technologies has resulted in the development of biological fungicides (or bio‐fungicides) that have been identified as sharing the second‐highest average market growth between 2012 and 2017, behind only the succinate dehydrogenase inhibitor fungicides (Phillips, 2020). Bio‐fungicides are products formulated with biological control agents that can act against soil‐borne and foliar fungal pathogens. They can be used alone or in combination with chemical fungicides (Ruano‐Rosa *et al*., 2018). These treatments have lower chances of resistance development and can reduce the doses of fungicides applied when compared with treatments with single fungicides, presenting great potential to contribute to IPM programs (Ons *et al*., 2020).

*Trichoderma* spp. are the most common biocontrol agents used as bio‐fungicides. They comprise about 60% of the efficacious bio‐fungicides worldwide. Their availability and dispersion are more widespread than commonly known, with the tendency to expand due to easier registration. More than 250 *Trichoderma*‐based products are available in the international market (Topolovec‐Pintarić, 2019). India represents the largest market for biological products formulated with *Trichoderma* spp., comprising about 90% of the Asian market. Next is Brazil, with the largest market in South and Central America (Woo *et al*., 2014; Topolovec‐Pintarić, 2019).

We expect that the development of effective bio‐fungicides, especially for the control of soil‐borne pathogens, has significant market potential. The chemical fungicides currently in use are either ineffective (e.g. in controlling *Fusarium* spp. and *Verticillium* spp.) or only partially effective (e.g. in controlling *Rhizoctonia* spp.) against soil‐borne pathogens that cause enormous crop loss annually. Due to the decline in the number of new fungicides in the market, any effective bio‐fungicide will have large market access and high adoption by both the organic and conventional farming as key part of IPM programmes.

## Key constraints of the agriculture microbial industry

Even though microbial products have an enormous potential to contribute to economic growth and sustainable development of agriculture, many challenges still limit the widespread global adoption of this technology. The limitations are often related to scaling up the efficacy of microbial products from controlled conditions, either in laboratory or greenhouse, to field conditions (Mitter *et al*., 2021). Even when a microbial product is successful in the field, results may not be consistent across different soils, crops or environments, limiting the wider adoption by the farmers (Naamala and Smith, 2020). The inconsistent efficacy in the field may be explained by the fact that the initial steps of microbial product tests, which are carried out in aseptic and controlled conditions, allow for an unbiased characterization of the microorganism in study (Mitter *et al*., 2021). However, when introducing these microbes to a complex agricultural setting, such as the field, several uncontrolled biotic and abiotic factors can influence the microbial inoculant success. For instance, indigenous microbial communities associated with the plant or with the soil can outcompete the introduced microbes. The inoculants can then disappear within weeks or persist at low and ineffective levels (French *et al*., 2021). Other factors such as extreme climatic conditions, poor soil characteristics and the presence of environmental and soil pollutants may also work against a strain which otherwise has excellent efficacy in controlled conditions. Some other practical challenges in the use of microbial inoculants include limited shelf‐life, incompatibility with agrochemicals or with the farmer’s equipment and production practices, lack of appropriate recommendations and issues related to storage and transportation (Santos *et al*., 2019; Mącik *et al*., 2020).

It is worth noting that the constraints limiting the wider use of microbe‐based products in agriculture vary greatly between developed and developing regions. Developed regions have scientific and commercial support, but the inconsistent efficacy of such products impacts their larger adoption. The farmers in these countries usually use the easily accessible agrochemicals to obtain more stable results (Naamala and Smith, 2020). In developing regions, such as some countries in Africa, the high costs, the lack of scientific support, low awareness or inadequate knowledge of such products, especially in small‐scale farms, has resulted in poor development of the sector. Inadequate regulatory frameworks and ineffective quality control systems are also contributors to the low adoption of microbial products in these regions (Raimi *et al*., 2021).

## Emerging solutions to critical limitations

**Fig. 1:**
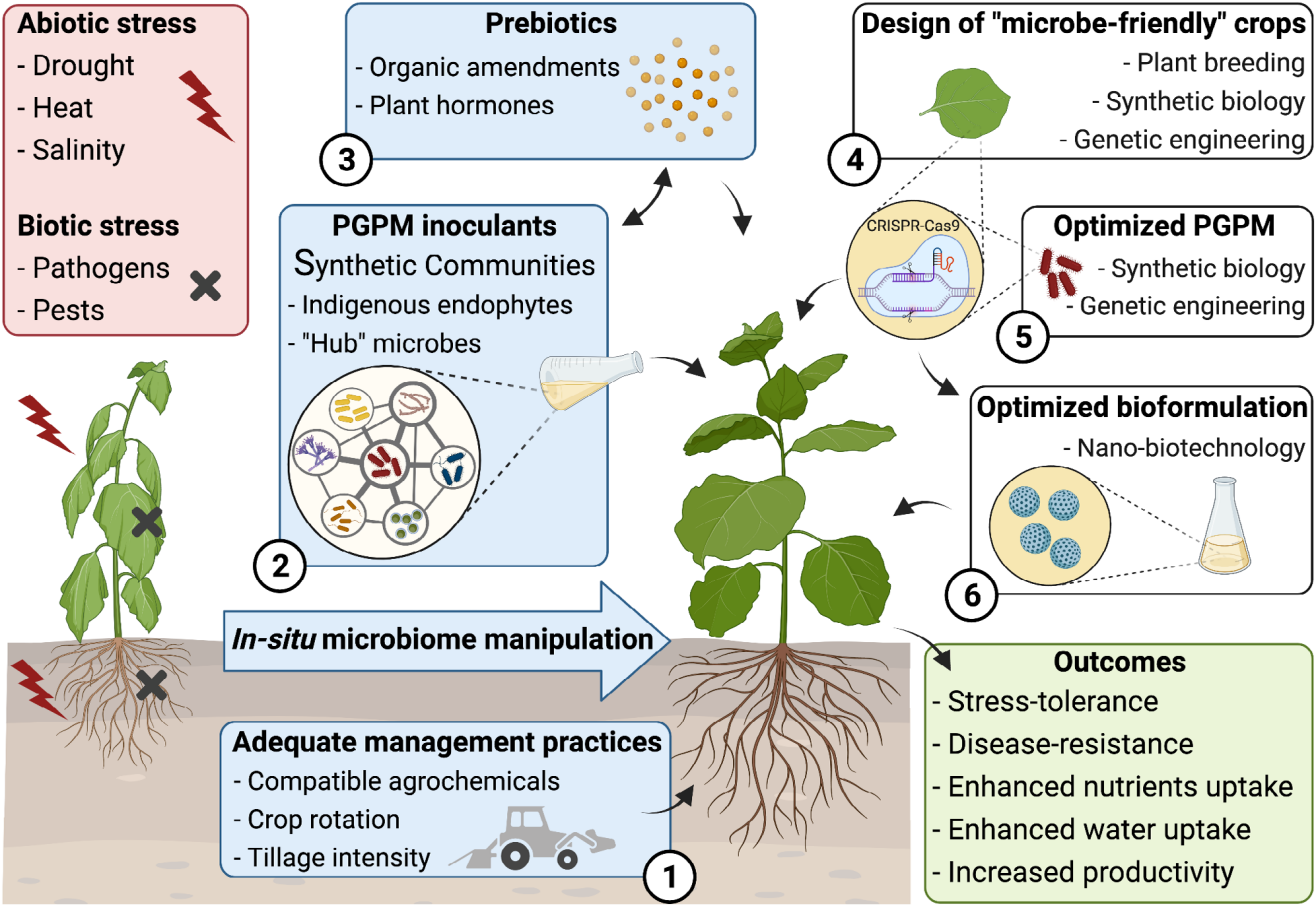
Traditional and emerging strategies to promote the establishment of beneficial plant–microbiome association and improve crop yield (created with BioRender.com). When microbial inoculants are introduced to the field, several uncontrolled biotic and abiotic factors (red box) can affect the inoculant’s success. To address this problem, the plant‐associated microbiome can be manipulated *in situ* to favour the establishment of plant‐beneficial microbes. Traditional *in situ* manipulation approaches (blue boxes) include the use of adequate management practices (1), the application of inoculants formulated with plant growth‐promoting microbes (PGPMs) (2) and the application of plant prebiotics (3). A new trend in the development of PGPM inoculants is the use of crop‐optimized synthetic communities containing indigenous and ‘hub’ microbes. Emerging strategies (white boxes), such as the design of ‘microbe‐friendly’ crops (4) and optimization of PGPM strains (5) and of bioformulations (6) are becoming available as areas such as genetic engineering/synthetic biology and nano‐biotechnology advance. In the near future, these emerging strategies are expected to be combined with traditional *in situ* microbiome manipulation approaches to expand the use of microbial tools in agriculture by tackling the issue of inconsistent efficacy.

To overcome the main constraints associated with the use of microbial products in agriculture, new strategies have been developed and are progressing rapidly (Fig. 1). The recent emergence of culture‐independent technologies such as next‐generation sequencing (NGS) to access and study microbiomes has opened opportunities to explore *in situ* microbiome manipulation approaches. This has allowed researchers to harness the plant‐associated microbiome without necessarily culturing microbial strains (Mueller and Sachs, 2015). One of the first steps in this approach is to define the core microbiome (i.e. microbial taxa that are consistently present in a plant species) associated with plants that are highly productive/healthy. This will help design practical ways to promote the establishment of the core microbiome aiming to improve crop yield.

*In situ* microbiome manipulation can be performed by ‘simply’ selecting the crop management practices that favour beneficial microbiomes (i.e. use of compatible agrochemicals, crop rotation and appropriate tillage intensity) (Hartman *et al*., 2018). Another traditional approach to manipulate microbiome *in situ* is the application of the aforementioned microbial inoculants formulated with PGPMs, aiming to increase the abundance of beneficial core microbes. There is a recent trend in the development of PGPM‐based products shifting away from inoculants containing a single microbial strain to products based on a consortium of microorganisms, or synthetic communities (SynComs). SynComs are usually designed to mimic, at some scale, the observed function and structure of the microbiome in natural conditions (de Souza *et al*., 2020). The main strategy is to tackle the issue of inconsistent efficacy by allowing a wider range of response to different environmental conditions and to competition with native microbial communities. In addition, SynComs can elicit a more broadly positive response in the inoculated plants (e.g. traits such as plant growth promotion and biological control can be combined). The fast‐evolving techniques applied to microbial ecology, genetics and microbiology are used to design these inoculants. The selection of key beneficial microorganisms to compose the SynComs can be based on the phylogeny or functional traits (genomically or experimentally deduced) of the microbes (Vorholt *et al*., 2017). Nevertheless, recent studies propose two key approaches to develop crop‐optimized SynComs by targeting and harnessing (i) the indigenous endophytic microbiota, aiming to introduce inoculants already adapted to the plant environment (Compant *et al*., 2021; Cain *et al*., 2020), with plant colonization ability and greater chances of survival (Qiu *et al*., 2019), and (ii) the core microbiota, with focus on the ‘hub’ microbes, which are those taxa that are highly connected or highly influential in the community (Toju *et al*., 2020). The need to use culturable microbes is still a limitation in using this approach. However, as culturable microbes will likely continue to be the main source of microbial inoculants in agriculture, at least from the short to the medium term, some strategies have been developed to address this problem. For example, some high‐throughput isolation and screening strategies aim to expand microbial culture collections, allowing for the cultivation of low abundance and rare taxa (Acuña *et al*., 2020).

The plant microbiome can also be manipulated *in situ* by applying organic soil amendments, which act as prebiotics by promoting growth or activity in a microbial community in a selective manner (Sheth *et al*., 2016; Arif *et al*., 2020). For instance, the application of broccoli residue and chitin amendment significantly reduced Verticillium wilt severity by enriching biocontrol agents in indigenous microbiomes associated with eggplants (Inderbitzin *et al*., 2018). There is also an emerging interest in the use of plant hormones to shape the plant‐associated microbiome and alleviate biotic and abiotic stressors. For example, amending soil with the ethylene precursor 1‐aminocyclopropane‐1‐carboxylate (ACC) can reshape the structure of soil microbiome, mitigating the impact of salinity on the soil and plant (Liu *et al*., 2019). Carvalhais *et al*. (2014) demonstrated that the plant hormones such as salicylic acid, methyl jasmonate, ethylene and abscisic acid can alter the composition of bacterial communities, which may influence plant productivity. As this area of research advances, new signalling molecules used in plant–microbe communication will be identified, and new pro‐ and prebiotics with enhanced efficacy will be developed. In our opinion, *in situ* microbiome manipulation has the greatest potential to transform the agricultural practices and sustainably increase crop productivity.

## Future outlooks

In the short to medium term, *in situ* microbiome manipulation strategies, such as the use of formulations containing SynComs and of new prebiotics, will likely dominate the development and commercialization of microbial tools in agriculture. In the future, the traditional strategies are expected to be combined with emerging technologies, such as synthetic biology and nano‐biotechnology (Fig. 1), aiming to tackle the issue of inconsistent efficacy and expand the use of microbial tools in agriculture.

Synthetic biology will allow to redesign the genome of plants and of PGPMs and even to improve the communication between them. The strategy in the plant‐mediated microbiome engineering approach is to obtain ‘microbe‐friendly’ crops. For instance, plants can be bred or genetically modified to release hormones or exudates that attract and maintain beneficial microbiomes (Arif *et al*., 2020). Gene editing tools such as CRISPR‐Cas9 (clustered regularly interspaced short palindromic repeats) and RNAi (RNA interference) have the potential to contribute towards these goals (Sudheer *et al*., 2020). Recently, CRISPR‐Cas9 has been used to elucidate genes involved in the mutual recognition between N‐fixing bacteria and legumes (Prabhukarthikeyan *et al*., 2020). For example, the gene *Rfg1*, responsible for restricting the nodulation by the N‐fixing *Sinorhizobium fredii* to specific soybean genotypes, was identified and validated using CRISPR‐Cas9 (Fan *et al*., 2017). The same tool was used to generate mutants of two PGPM (*Bacillus subtilis* HS3 and *Bacillus mycoides* EC18), with potential relevance for their biocontrol abilities (Yi *et al*., 2018). Although the CRISPR‐Cas9 approach has provided only initial insights into the molecular basis of the plant–microbe interaction, it will likely assist in developing sustainable strategies for agriculture in the future. It should be noted that the use of genetically modified organisms in agriculture is limited and remains an extremely controversial topic. Issues linked to public perception and regulatory requirements need to be addressed for wider adoption of genetically modified organisms in agriculture.

Many limitations that prevent the introduced microbe(s) to achieve the desired effects in plant growth and fitness can also be overcome by the development of appropriate bioformulations. A bioformulation is composed of selected microbial strain(s) and an inert carrier material, which should maintain the viability and stability of the microbial cells during production and distribution (Rani and Kumar, 2019). Different bioformulations have been developed using liquid or solid materials as carriers. Recent reviews give a comprehensive overview on bioformulation development, potential constraints and future trends (Berninger *et al*., 2018; Sahai *et al*., 2019; Chaudhary *et al*., 2020). Importantly, bioformulations should be cost‐effective, compatible with the existing production practices and withstand harsh environmental/storage conditions (Vassilev *et al*., 2020). Nano‐biotechnology is considered as one of the key technologies in the twenty‐first century and offers many potential applications to improve the delivery and stability of bioformulations. Nanoparticles (NPs) can be employed to deliver PGPMs and their derived compounds in a regulated manner, that is, focussing on specific types of cells or tissues, at specific times (Timmusk *et al*., 2018). In addition, nanoencapsulation technology could be used to protect biofertilizer (and biopesticide) components, enhancing their shelf‐life and controlling their dispersion (Vejan *et al*., 2016). Many PGPMs treated with gold‐, aluminium‐ and silver‐coated nanoparticles have been reported to significantly increase plant growth and to inhibit pathogen growth (Gouda *et al*., 2018; Kumari and Singh, 2020). Recent studies have shown that the use of biocompatible titania and silica nanoparticles promoted the attachment and colonization of PGPMs, which resulted in plant biomass accumulation and growth improvements in stressful conditions (Timmusk *et al*., 2018; Fetsiukh *et al*., 2020). The development of nano‐formulations containing Bt (or Bt‐derived compounds) also holds great potential in increasing the shelf‐life, efficacy and persistence in the field of these biopesticides (Devi *et al*., 2019). The development of nano‐biofertilizers and nano‐biopesticides will certainly be a step ahead in the innovative field of sustainable agriculture (Kumari and Singh, 2020). However, issues related to health and environmental toxicity of NPs will need to be properly addressed before the large‐scale application and commercialization of such technology.

## Concluding remarks

Plant growth‐promoting microbes are expected to sustainably boost agricultural production over the next few decades. Although microbial tools have been used in agriculture for over 120 years, their full potential is only now being explored as a result of recent technological advances. Although the sector is still in its infancy, the path towards effective microbial products is taking shape and their market is growing rapidly. Despite the increasing demands from consumers and policymakers and the many stories of success, much remains to be accomplished, in particular overcoming inconsistent efficacy in diverse field conditions. This includes complex fundamental and translational research questions (Box 1), which must be addressed to allow the development of efficient microbial tools in agriculture.

Box 1 **Fundamental and translational research questions to be addressed for the development of microbial tools in agriculture**

**Fundamental research questions**

- What are the eco‐evolutionary processes that govern plant–microbiome interactions?
- What are the taxa that constitute the core and hub microbiota of a crop species?
- What are the key communication pathways between plant and microbiome?
- What are the main microbe–microbe interaction pathways within the plant microbiome?

**Translational research questions**

- What are the colonization requirements of the introduced microbial inoculant?
- Do microbial inoculants have competitive advantage over indigenous microbiomes?
- What are the main processes that improve inoculant colonization?
- What are the requirements of formulation and viability during storage and transportation?
- What is the best time and mode of application of a microbial inoculant?

Going forward, clear communication about the requirements for research and the time needed to deliver the product to the market is essential. It is important to properly manage the expectations of both consumers and industry to avoid frustration and the early termination of promising strategies. Unreasonable expectations, particularly in timeline and resources required from discovery to commercial application, impact significantly this emerging industry. This can lead to reduced investment and public interest, which will ultimately reduce both policy support and adoption by farming communities. At least in the short to the medium term, biofertilizers and biopesticides should not be viewed as a complete replacement for chemical fertilizers and pesticides but as a component of an integrated nutrient/pest management strategy.

The success of microbial tools in agriculture is currently measured in economic gain, either through reaching increased yields or reducing the application of agrochemicals, or both. An explicit effort to combine economic, environmental and social benefits can also help increase the adoption of such products. This is particularly relevant in developed countries where environmental and social benefits can be encouraged by the governments.

On the other hand, developing countries, where agriculture is the main driver of the economy and livelihood, have the greatest potential to benefit from the exploitation of beneficial plant‐associated microbes. Microbial inoculants may play a significant contribution to these regions as they can be produced locally by small companies and can be used in small agricultural farms. Additionally, they offer a potential solution for the high cost of agrochemicals in these regions and can create new jobs and regional economic growth with positive social outcomes.

Overall, a systematic and concerted effort from all key stakeholders (e.g. researchers, policymakers, manufacturing industry and farming community) will be required for the widespread global use of microbial products in agriculture. Further public–private partnerships, multidisciplinary approaches and long‐term investments with realistic timelines and goals are critical for this industry growth. Altogether, these approaches will support the adoption of microbial tools as a standard agricultural practice worldwide, contributing simultaneously to achieve food security, environmental sustainability and climate change mitigation.

## Funding Information

Plant microbiome research in BKS laboratory is supported by the Australian Research Council (DP190103714; DP210102081) and Cotton Research and Development Corporation.

## Conflict of interest

None declared.
